# Carotid Interventions in Patients Undergoing Coronary Artery Bypass Grafting: A Narrative Review

**DOI:** 10.3390/jcm13113019

**Published:** 2024-05-21

**Authors:** Andrea Xodo, Alessandro Gregio, Fabio Pilon, Domenico Milite, Tommaso Hinna Danesi, Giovanni Badalamenti, Sandro Lepidi, Mario D’Oria

**Affiliations:** 1 Vascular and Endovascular Surgery Division, “San Bortolo” Hospital, AULSS8 Berica, 36100 Vicenza, Italy; 2 Division of Cardiac Surgery, Brigham and Women’s Hospital, Boston, MA 02115, USA; 3 Vascular and Endovascular Surgery, Department of Clinical Surgical and Health Sciences, University of Trieste, 34149 Trieste, Italy

**Keywords:** coronary artery disease, carotid artery stenosis, coronary artery bypass, carotid endarterectomy, carotid artery stenting

## Abstract

Simultaneous carotid artery stenosis (CS) and coronary artery disease (CAD) is a common condition among patients with several cardiovascular risk factors; however, its optimal management still remains under investigation, such as the assumption that carotid disease is causally related to perioperative stroke and that preventive carotid revascularization decrease the risk of this complication. Synchronous surgical approach to both conditions, performing carotid endarterectomy (CEA) before coronary artery bypass graft (CABG) during the same procedure, should still be considered in selective patients, in order to reduce the risk of perioperative stroke during coronary cardiac surgery. For the same purpose, staged approaches, such as CEA followed by CABG or CABG followed by CEA during the same hospitalization or a few weeks later have been described. Hybrid approach with carotid artery stenting (CAS) and CABG can also be an option in selected cases, offering a minimally invasive procedure to treat CS among patients whom CABG cannot be postponed. When carotid intervention is indicated in patients with concomitant CAD requiring CABG, a personalized and tailored approach is mandatory, especially in asymptomatic patients, in order to define the ideal surgical strategy. The aim of this paper is to summarize the current “state of the art” of the different approaches to carotid artery diseases in patients undergoing CABG.

## 1. Introduction

Atherosclerosis is generally viewed as a progressive and chronic inflammatory process that can affects different vascular districts. In fact, given its intrinsic systemic nature, severe atherosclerosis usually concerns (although with varying degrees of severity) the brain, the coronary arteries and peripheral circulation or any combination thereof [1].

Regarding cerebrovascular disease, stroke is a major cause of death and disability worldwide and approximately a quarter of ischemic strokes result from embolic events related to carotid artery stenosis (CS), in particular for severe lesions (stenosis levels of 80–99%) [2].

It is well known that atherosclerosis is a chronic inflammatory disease of large and medium-sized arteries, which involves the formation of degenerative changes in the arteries called atherosclerotic plaques, gradually narrowing the vessel lumen, which may result in ischemia of the organ supplied. It should be noted that carotid revascularization may be surgical (endarterectomy) or percutaneous (angioplasty and stenting). Especially in the case of percutaneous procedures (and less so for surgical procedures), the phenomenon of restenosis remains a significant problem, which may limit the effectiveness of such a procedure and result in the need for reintervention (although criteria for secondary procedures are usually more stringent).

The prevalence of atherosclerotic lesions of carotid arteries with a stenosis > 50% is present in 9% of patients undergoing coronary artery bypass graft (CABG), while the prevalence of severe CS > 80% is evaluated at around 7% [3]; these conditions may be associated with an increased risk of ischemic stroke during and after cardiac surgery [4,5]. Overall, the incidence of stroke after CABG is 1 to 5% and it carries a significant impact on mortality risk during the first year compared with patients without stroke [6,7].

Perioperative cerebral infarction after cardiac operations can be attributed to several factors including preexisting vascular disease, transient hypercoagulability, procedure-related thromboembolism, micro- and/or macro-embolism and systemic inflammatory response syndrome (SIRS) or a combination of these factors.

Conventional on-pump coronary artery bypass grafting (OP-CABG) remains the gold standard for surgical coronary artery revascularization [8]. However, this technique is often associated with adverse effects such as SIRS, renal or neurological dysfunction and other postoperative complications [9].

Furthermore, high mean arterial pressure (MAP) may increase the risk of postoperative cognitive deficit (POCD) after cardiopulmonary bypass (CPB) surgery, due to the higher bleeding at the surgical site, the need for cardiotomy suction as well as the risk of embolic load [10,11]: there are therefore a variety of ways, at least potentially, in which the brain may be injured during a cardiac surgical procedure with CPB [12].

**Figure 1:**
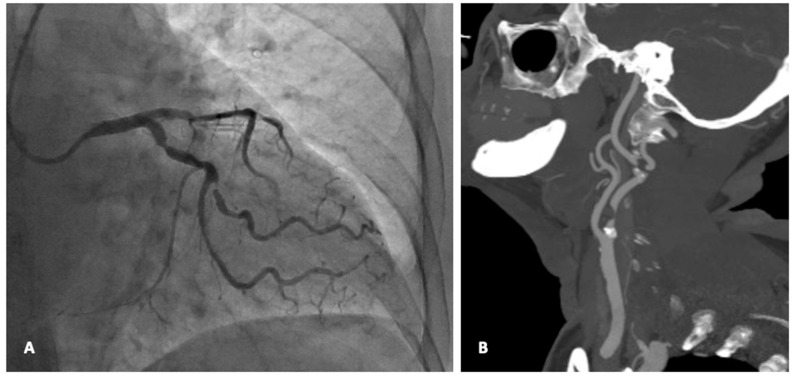
A 75 female with severe multivessel coronary disease and pre-occlusive stenosis of the right ICA (**Panel A**), who underwent synchronous CEA with patch angioplasty and CABG (**Panel B**). (From the Authors’ own collection, reproduced with patients’ permit).

**Figure 2:**
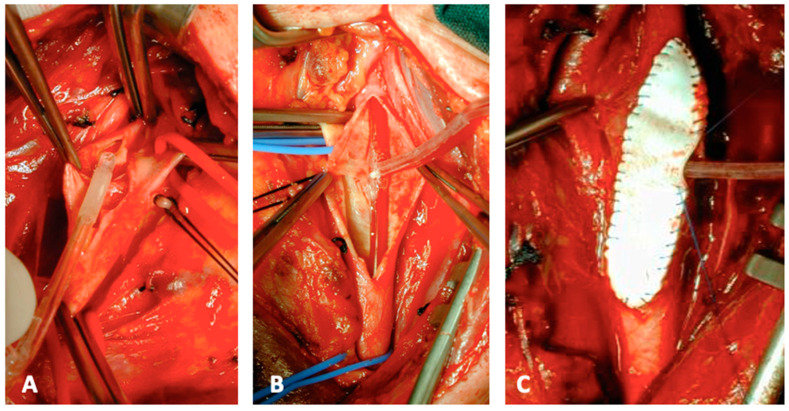
Intraprocedural steps of CEA: Pruitt-Inahara shunt insertion (**A**,**B**). Shunt insertion allows the completion of the patch closure in case of a straight ICA (**C**). (From the Authors’ own collection, reproduced with patients’ permit).

Synchronous open surgical revascularization, in particular carotid endarterectomy (CEA; Figure 1, Figure 2) followed by CABG during the same procedure, has been proposed and preferred by many surgeons, in order to reduce the risk of neurological events.

However, other options including a staged approach (CEA followed by CABG or CABG followed by CEA during the same hospitalization or a few weeks later) or hybrid procedures like carotid artery stenting (CAS) before CABG, can be considered to optimize the peri-operative risk-benefit ratio for the brain and for the heart.

Despite the relative abundance of publications on this topic, no randomized controlled trials (RCTs) exist, and practice patterns may vary according to physicians’ and/or institutions’ preferences. 

Indeed, optimal surgical management of concurrent CS and coronary artery disease (CAD) has been a matter of discussion, favored by scarce and frequently contrasting results in the literature.

The aim of this narrative review was to investigate the current “state of the art” of the approach to the treatment of CS in patients undergoing CABG and the potential risk-benefit ratio of different available procedures.

## 2. Methods

A non-systematic search of the literature was conducted from the PubMed and Scopus databases. They were searched to identify relevant English-language articles fully published in the period 1 January 2010–1 January 2023.

The following MeSH search terms were used: stroke, cerebrovascular ischemia, CABG, CEA, carotid stenosis, carotid stenting, CAS. These were adopted in combination with the Boolean operators AND or OR. Reference lists of selected articles were also searched. Editorials, case reports with <5 cases, conference abstracts and non-English-language articles were excluded. The review was constructed following the Scale for the Assessment of Narrative Review Articles principles [13].

### 2.1. Peri-Operative Risk of Stroke and the Link with Carotid Stenosis: “To Screen or Not to Screen Carotid Arteries before CABG?”

Stroke is an important cause of mortality and morbidity after cardiac surgery, with different aetiologies; however, a clear direct causal relationship between CS and ipsilateral stroke is often missing in more than 75% of patients with brain ischemia after CABG [14].

Different institutions routinely perform carotid artery duplex ultrasound (DUS) screening before CABG, with the expectation of identifying patients with significant CS and reducing their perioperative neurologic events; moreover, the clinical utility and cost-effectiveness of carotid DUS prior to cardiac surgery have been questioned [15].

Current 2023 ESVS guidelines do not recommend routine screening for CS in CABG candidates asymptomatic for cerebrovascular symptoms, and more in general for patients undergoing open heart surgery (Rec. n. 109, Class III Level C) because it has no significant impact in reducing perioperative neurological complications [3].

Similar recommendations are reported in the 2018 ESC/EACTS Guidelines, with limited indications for preoperative carotid bifurcation screening with DUS in patients undergoing CABG (a strong evidence is reported only for patients with recent history of stroke or TIA, Class I Level B) [16].

In spite of this, a selective screening protocol for elderly patients older than 70 years old or in the presence of previous neurological symptoms (history of TIA or stroke), carotid bruit, multivessels CAD or concomitant peripheral artery disease (PAD) could be considered to identify a subgroup of patients at higher risk for stroke due to diffuse atherosclerosis (including for the aorta), that could benefit from strategies to minimize aortic arch manipulation during CABG [3,16,17,18].

Nevertheless, the Society for Vascular Surgery (SVS) clinical practice guidelines for management of extracranial cerebrovascular disease, published in January 2022, find “appropriate” a screening for asymptomatic CS in patients undergoing CABG, considering the prevalence of occult carotid disease in patients with CAD [19,20].

Risk prediction models may be useful tools to detect individuals “at high risk”, allowing improved cardiovascular risk management to prevent cardiovascular complications and selective screening in these patients [21]. Regarding patients who have recently presented neurological events (hemi-spheric neurologic symptoms, amaurosis fugax, TIA or stroke), they must be evaluated with carotid DUS before CABG to exclude or to detect a carotid artery stenosis; findings of moderate or severe stenosis should be confirmed on computed tomography angiography (CTA).

### 2.2. Stroke and Neurological Complications during and after CABG: A Focus on the Preoperative, Intraoperative and Postoperative Risk Factors

Notably, CS is not the only factor that could play a role in the development of stroke after coronary artery cardiac surgery [22,23]. In fact, as well documented for other vascular procedures like thoracic endovascular aortic repair (TEVAR), stroke is a multifactorial procedure-related complication [24].

To better understand all the involved variables, it’s useful to classify post-operative neurological events according with time of onset.

(A) Preoperative risk factors

Different risk factors associated with perioperative stroke have been identified, including age, previous stroke, arteriosclerotic burden and PAD [25,26]. Most of these risk-factors are “non-modifiable”, making necessary to carefully select patients before surgery and defining the best surgical strategy.

Magedanz and colleagues have developed a risk score model for stroke after cardiac surgery (however including also patient treated for valve replacement and not only for CABG): in this study, only one factor may be considered “modifiable” (CPB time > 110 min), while age, emergency procedure, PAD and history of cerebrovascular disease were “non-modifiable” independent predictors of stroke in the post-operative period [27].

Regarding medical therapy “critical issues”, continuing aspirin before CABG is not associated with a significantly increased risk of all-cause mortality, perioperative myocardial infarction or postoperative bleeding, and instead, aspirin has been proved being efficient in reducing the incidence of ischemic events, improving venous graft patency and survival rate after CABG [28,29].

(B) Intraoperative risk factors

There are several factors associated with intra-operative strokes during CABG, including manipulation of the ascending aorta or of the arch during aortic cannulation and aortic clamping (with the risk of embolic stroke), the use CPB or a low cerebral perfusion pressure during the procedure [30].

Coronary revascularization performed under cardioplegic arrest relies on aortic cannulation and manipulation and aortic cross clamping, with a non-negligible risk of athero-embolisation [31].

An off-pump coronary artery bypass grafting (OFP-CABG) technique, avoiding all these maneuvers, carries a significant neurologic advantage, especially in patients with a previous TIA or stroke, and is recommended by the latest 2021 American Coronary Revascularization Guidelines in cases of ascending aortic disease [32,33].

Furthermore, it was shown that microemboli, defined by transcranial duplex (TCD) in the middle cerebral arteries during cardiac surgery, are associated with neurological injuries after CABG, however without identifying a direct link between large numbers of macrobubbles and adverse cognitive outcomes [34].

Also intracranial cerebral arterial disease has been detected as an independent risk factor for central nervous system complications of CABG surgery, suggesting pre-CABG evaluation of the cerebral arteries by TCD for the risk assessment of these procedures [35].

(C) Postoperative risk factors

Perioperative stroke is defined as stroke occurring within 30 days following surgery. In the early post-operative period (within seven days) the likeliest cause of stroke is represented by dysrhythmias, in particular atrial fibrillation, that may lead to systemic embolism [36].

Low cardiac output syndrome, characterized by an inadequate cardiac pump function resulting in reduced oxygen delivery and hypoxia, may be considered another cause of stroke after CABG.

Moreover, a non negligible percentage of covert stroke has been identified by Mrkobranda and colleagues in patients who completed a brain magnetic resonance imaging after CABG surgery: in fact, 19 patients (39% of the cohort) presented a covert stroke, while 3 patients (6%) had a clinical stroke within 30 days of surgery [37].

### 2.3. Is Carotid Artery Disease Responsible for Stroke after CABG?

A correlation between the severity of the carotid stenosis and risk of neurological events is well documented in the literature. Particularly, the presence of a symptomatic carotid stenosis, a contralateral carotid artery occlusion or a bilateral 70–99% stenosis were found to significantly increase the stroke risk: despite these findings, no definitive statement on the existence of a causal relationship between CS and perioperative stroke during and after coronary artery bypass surgery could be made. 

Nevertheless, the increase in neurological complications observed in patients with bilateral severe CS or occlusion may be a manifestation of cerebral hypoperfusion, exacerbated by CPB, but also a sign of a more diffused and advanced atherosclerotic disease involving the aortic arch. 

If a causal relationship is implicated, previous or simultaneous carotid artery intervention should therefore yield a lower risk of neurological complications, but current literature does not support this hypothesis. 

A recent meta-analysis by Tsukagoshi et al. evaluated outcomes between different strategies of carotid and coronary artery revascularization compared with CABG alone. No statistically significant differences were found between the two groups in either perioperative stroke or perioperative mortality [38].

Similarly, a recent RCT randomized 127 patients with severe asymptomatic carotid stenosis to either simultaneous CABG + CEA vs. CABG alone. Both at 30 days and at five years of follow-up no significant differences were found in the rates of non-fatal stroke and combined non-fatal stroke + death [39].

### 2.4. How to Reduce Stroke and Relevant Neurological Complications during CABG? The Point of View of Cardiac Surgeons

Strategies to reduce manipulation of the aortic arch during CABG could prove useful in the reduction of stroke rates, especially in patients with so-called “porcelain aortas” or “shaggy aortas” in whom there is a higher atherothrombotic burden within the aorta. 

Borger and colleagues performed a large retrospective study on 6682 patients underwent CABG, identifying that the majority of strokes (37%) during CABG were caused by cerebral macroemboli, presumably from the ascending aorta (AscAo) [40].

The discernment of macroemboli as the principal cause of stroke is important for different reasons: first of all, in order to identify high risk patients. Diffuse atherosclerosis of the AscAo can be detected by computed tomography (CT) or transesophageal echocardiography and in these patients adjunctive procedures (intra-aortic filters or alternative aortic cannulation techniques) can be adopted to minimize the stroke risk [40,41].

Several techniques and strategies have been developed to reduce the risk of stroke during CABG. These include single clamp technique for proximal anastomoses, off-pump surgery with proximal anastomotic devices, and totally arterial revascularization with composite grafts. 

Patients presenting porcelain aorta might benefit from hybrid revascularization strategy including left internal mammary artery (LIMA) to left anterior descending artery (LAD) and or LIMA to LAD plus Y conduit for a second and a third target, and staged percutaneous coronary intervention (PCI) for targets have not been surgically addressed [42].

The surgical portion of an hybrid revascularization can be performed both on- and off-pump or with Impella (Abiomed, Danvers, MA, USA) support [43].

In a subset of patients with severe CS, Naito et al. have found a high postoperative rate of neurological complications after concomitant CEA and CABG with aortic cross-clamp compared to CEA and CABG performed without cross clamping (21% vs. 0%), suggesting that cross clamping, rather than the presence of a CS stenosis, is responsible for the majority of perioperative neurological events [44]. Similarly, Kowalewski et al. in a meta-analysis involving 19.192 subjects found a significant reduction in the odds of cerebral stroke comparing standard CABG with off-pump CABG (OR 0.72; 95% CI 0.56–0.92; *p* = 0.009) [45].

### 2.5. Guidelines Reccomendation Regarding Treatment of Carotid Stenosis in Patients Undergoing CABG

The 2023 ESVS guidelines recommend treatment by staged or synchronous carotid endarterectomy in patients with 50–99% carotid stenosis and a history of stroke or TIA in the preceding 6 months (Class IIa, level of evidence B). With regards to asymptomatic stenosis, carotid intervention by staged or synchronous CEA or CAS may be considered in case of a bilateral stenosis > 70% or a stenosis > 70% with a contralateral occlusion (Class IIb, level of evidence C) [3].

In a similar way, Society for Vascular Surgery (SVS) guidelines recommend staged or synchronous carotid interventions in patients who require CABG and present a 50–99% CS and a history of TIA or stroke in the preceding 6 months (grade 2C). With the same weight of evidence, CEA is suggested before or concomitant with CABG in patients with severe bilateral CS or severe asymptomatic carotid stenosis associated with a contralateral occlusion [19].

ESC/EACTS Guidelines suggests a prophylactic carotid revascularization only for “high risk” patients (severe bilateral lesions or prior stroke/TIA), after a multidisciplinary discussion that should include a neurologist [16].

### 2.6. What Is the Best Strategy to Treat Carotid Stenosis in Patients Undergoing CABG?

As noted before, a variety of different strategies have been described to manage CABG candidates with concomitant carotid stenosis. Each of these strategies comes with their own advantages and shortcomings, and the choice of treatment should be tailored to the individual patient and clinical scenario. In the section below, we provide a comprehensive yet succinct overview of the available strategies, discuss their risks and benefits, as well as provide key supporting evidence (Table 1).

Management options include:

- synchronous CEA + CABG (CEA procedure is performed first, followed by CABG during the same operation).
- staged CEA + CABG.
- staged CABG + CEA (at least one or more days of delay between the two procedures).

hybrid approaches (CAS associated with CABG, synchronous or staged)

(A) Synchronous CEA + CABG

In presence of coexisting critical carotid and coronary artery disease, treating both districts at one stage is the most common revascularization approach described in the literature. 

CEA could be performed either prior or during cardiopulmonary bypass (CPB) time: however, performing CEA before CPB seems to offer better outcomes in term of perioperative morbidity than a combined CEA/CABG using CBP for both procedures, however with a similar rate of neurological complications [46].

According to a recent meta-analysis by Tsukagoshi et al. outcomes regarding perioperative stroke after “standard” staged procedure (CEA followed by CABG) are significantly better if compared to reverse staged procedures (CABG and then CEA), with an OR of 0.41 [95 CI 0.23–0.74; *p* = 0.003]. No significant differences with other strategies were found for perioperative mortality/myocardial infarction (MI) and bleeding complications [38].

Synchronous interventions are carried out under general anesthesia, with continuous EEG monitoring, to check shunt functioning throughout the procedure and to monitor the cerebral status. EEG use is important during CEA + CABG, because post-operative neurological status could not be usually evaluated until several hours after CEA and this adjunctive tool gives further importance to intraoperative neurological monitoring and quality control. 

Furthermore, in our experience, routine shunting with delayed insertion after plaque removal is usually performed, independently from electroencephalographic modifications. This shrewdness seems to be safe and effective and contributes to maintain a low neurological complication rate, especially in patients with a recent stroke or TIA [47].

Modified carotid artery revascularization techniques are effective alternatives to standard CEA, however these should be performed only in selected cases, due to the higher rate of global central neurological complications (considering together TIA, minor stroke and major stroke) compared with patients treated with standard CEA [48].

A technical measure to mitigate bleeding complications or a formation of neck haematoma involves deferring surgical wound closure of the CEA incision after the completion of CABG, to check for potential bleeding following systemic heparinization for cardiopulmonary bypass.

(B) Staged CEA and then CABG

Conducting CEA prior to CABG may be advisable for patients with stable angina and symptomatic carotid stenosis. Perioperative stroke and mortality rates are comparable to those of synchronous CEA + CABG [36]. Nevertheless, this approach suffered from numerically worse results in terms of perioperative MI when compared with alternative strategies. Performing CEA as the first step in patients with relevant coronary artery disease could increase risk of postoperative MI and explain the tendency towards worst outcomes in this regard.

(C) Staged CABG and then CEA

This approach is rarely described in literature. It could be applied particularly in case of unstable cardiac symptoms and concurrent asymptomatic CS. When compared with synchronous or staged CEA > CABG interventions, it was associated with significantly more perioperative strokes [29].

(D) Hybrid approach: synchronous CAS + CABG

As a minimally invasive procedure associated with a lower cardiovascular risk, CAS could prove particularly useful for treatment of CS in CABG candidates. 

When compared to strategies with CEA, CAS + CABG was associated with lower perioperative rate of mortality/MI, but a significantly higher rate of perioperative stroke/TIA [29].

As current guidelines and data from RCT showed, CAS should be avoided in symptomatic CS due to the higher risk of perioperative neurological complications. 

This consideration remains valid even in the case of a patient candidate for CABG, as demonstrated by Paraskevas et al., who found a high rate (15%) of neurological complications in symptomatic patients undergoing CAS + CABG. The same meta-analysis compared different timing strategies for carotid artery stenting and CABG. No significant difference was found comparing outcomes of same-day CAS + CABG and staged CAS + CABG [49].

Trans-carotid artery revascularization (TCAR) is an alternative to typical trans-femoral CAS and may be considered in patients candidate to endovascular treatment of a CS in presence of “shaggy” aortic arch, hostile supra-aortic trunks cannulation or complex femora/iliac access. 

TCAR is meant to decrease the risk of micro-embolic stroke by reversing flow in the carotid artery during stenting.

Recently, TCAR with flow reversal performed as the same time as the CABG has been proposed as an alternative method of carotid artery revascularization. The procedure, as described by Williams et al. in their initial experience, require exposure of the common carotid artery in the mediastinum via the sternotomy and insertion of the arterial sheath into the CCA after proximal clamping. Flow reversal is obtained by a percutaneous access to the femoral vein. Then, a self-expanding stent is placed in the usual fashion [50].

(E) Hybrid approach: staged CAS + CABG

CAS followed by CABG present the theoretical advantage to treat with CABG a patient recently treated for a CS with a minimally invasive procedure. 

Moreover, the advantages of staged CAS followed by CABG include the avoidance of general anesthesia, which may reduce cardiac complications.

A prospective study conducted on 112 patients underwent CABG without carotid artery intervention and 62 patients who were scheduled for CAS + CABG: the risk of ipsilateral stroke in group of patients treated with isolated CABG was lower compared with group of patients treated with CAS + CABG. However, these patients presented a higher prevalence of a history of neurological event at any time, cerebrovascular symptoms, or bilateral CS [51].

A particular and relatively rare condition may be represented by a significant lesion of the ostium of the supra-aortic trunks (in particular the innominate artery or the left common carotid artery) associated with a significant CS and CAD: for these cases, a combined surgical approach may be evaluated [52]. Alternatively, a hybrid approach with stenting of the supra-aortic main trunk(s) before CABG may be a suitable option [53].

### 2.7. General Considerations on Antiplatelet Therapy

The need for Dual Antiplatelet Therapy (DAPT) during and after CAS may pose a problem for bleeding complications during CABG. In fact, CAS patients undergoing CABG are suspected to be at in-creased risk of bleeding complications because of DAPT (acetylsalicylic acid indefinitely and clopidogrel for at least a month after CAS) [54].

Paraskevas et al. analyzed outcomes of five different antiplatelet strategies in a meta-analysis of patients with concurrent carotid and cardiac disease who underwent CAS followed by CABG [49].

Despite significant heterogeneity between studies, the main observation was that stopping DAPT prior to CABG seemed to be associated with high risk of death/stroke (13.4%) and death/stroke/MI (14.4%). With respect to bleeding complications, a single antiplatelet regime until CABG, followed by DAPT after CABG had the highest rate of chest reopening for bleeding (5.9%) [49].

Although definitive conclusions cannot be drawn from the literature, the safest solution may be to perform CAS with a single antiplatelet agent and introduce the second antiplatelet agent when deemed surgically safe, following CABG.

### 2.8. Study Limitations

The present paper should be interpreted in the context of several limitations. In particular, for the nature of the study (narrative review), it presents inherent limitations in term of objectivity, completeness of literature search and interpretation of findings. Lastly, we did not address the issue of how to address post-CABG stroke (in terms of diagnosis, treatment, and prognosis) as this remained beyond the topic of the study despite being a clinically relevant consideration.

## 3. Conclusions

Carotid revascularization is a safe and effective treatment for patients with concomitant carotid and coronary artery disease, and may be justified in symptomatic and high-risk patients with contralateral carotid occlusion or bilateral CS.

Stroke after CABG carries a significant impact on patients’ mortality and morbidity, however the main mechanism of this devastating complication after CABG is represented by atheroembolization, in particular from the aortic arch [55]; strategies for prevention include medical therapy, preoperative evaluation of preexisting risk factors (including aortic atherosclerosis and carotid stenosis) and intraoperative adjunctive techniques.

Different approaches are available nowadays for the treatment of CS in patients undergoing CABG, outlining the importance of a tailored approach and determining proper indications for carotid intervention. A multidisciplinary and dedicated team should play a central role in the treatment of this broad array of complex and multivessel disease.

Future research will be needed to address outstanding issues such as the best revascularization strategy and optimal timing with coronary surgery, the appropriate level of case-volume and the impact of newest surgical (open and endovascular) and anesthesiologic techniques, the adoption of machine learning algorithms for identification of “high-risk for stroke” patients, and the implementation of more standardized approaches for postoperative management and neurological evaluation.

## Figures and Tables

**Table 1:** Advantages and disadvantages of the different approaches to the combined treatment of carotid stenosis using CEA/CAS and coronary arterial disease using CABG.

Procedures	Advantages	Disadvantages
Synchronous CEA + CABG	- Single procedure - Same anesthesia	CEA then CABG - Difficult to check neurological status after CEA. - Partial emostatic control after CEA. CABG then CEA - Risk of intrathoracic bleeding during CEA - No benefit in term of reduction of neurological injuries during CABG
Staged CEA and then CABG	- CABG performed with a lower risk of neurological injuries	- Two different procedures - Risk of acute coronary syndrome in the “waiting time” between CEA and CABG
Staged CABG and then CEA	- CEA performed with a lower risk of cardiac adverse events	- Two different procedures - Risk of acute cerebrovascular event in the “waiting time” between CABG and CEA
Hybrid approach: - Synchronous CAS + CABG - Staged CAS and then CABG	- Low invasive procedure in term of carotid intervention	- Preferably needs of hybrid room. - DAPT needed after CAS

## Data Availability

The research did not involve original data but only review of published literature.

## References

[1] Achim A., Péter O., Cocoi M., Serban A., Mot S., Dadarlat-Pop A., Nemes A., Ruzsa Z. (2023). Correlation between Coronary Artery Disease with Other Arterial Systems: Similar, Albeit Separate, Underlying Pathophysiologic Mechanisms. J. Cardiovasc. Dev. Dis..

[2] Högberg D., Björck M., Mani K., Svensjö S., Wanhainen A. (2019). Five Year Outcomes in Men Screened for Carotid Artery Stenosis at 65 Years of Age: A Population Based Cohort Study. Eur. J. Vasc. Endovasc. Surg..

[3] Naylor R., Rantner B., Ancetti S., de Borst G.J., De Carlo M., Halliday A., Kakkos S.K., Markus H.S., McCabe D.J., Sillesen H. (2023). Editor’s Choice—European Society for Vascular Surgery (ESVS) 2023 Clinical Practice Guidelines on the Management of Atherosclerotic Carotid and Vertebral Artery Disease. Eur. J. Vasc. Endovasc. Surg..

[4] Mao Z., Zhong X., Yin J., Zhao Z., Hu X., Hackett M.L. (2015). Predictors associated with stroke after coronary artery bypass grafting: A systematic review. J. Neurol. Sci..

[5] Naylor A.R., Mehta Z., Rothwell P.M., Bell P.R.F. (2002). Carotid artery disease and stroke during coronary artery bypass: A critical review of the literature. Eur. J. Vasc. Endovasc. Surg..

[6] Stamou S.C., Hill P.C., Dangas G., Pfister A.J., Boyce S.W., Dullum M.K., Bafi A.S., Corso P.J. (2001). Stroke after coronary artery bypass: Incidence, predictors, and clinical outcome. Stroke.

[7] Wagner B.D., Grunwald G.K., Almassi G.H., Li X., Grover F.L., Shroyer A.L.W. (2020). Factors associated with long-term survival in patients with stroke after coronary artery bypass grafting. J. Int. Med. Res..

[8] Anyanwu A.C., Adams D.H. (2018). Total Arterial Revascularization for Coronary Artery Bypass: A Gold Standard Searching for Evidence and Application. J. Am. Coll. Cardiol..

[9] Hillis L.D., Smith P.K., Anderson J.L., Bittl J.A., Bridges C.R., Byrne J.G., Cigarroa J.E., Disesa V.J., Hiratzka L.F., Writing Committee Members (2011). 2011 ACCF/AHA Guideline for Coronary Artery Bypass Graft Surgery: A report of the American College of Cardiology Foundation/American Heart Association Task Force on Practice Guidelines. Circulation.

[10] Cheung A.T., Messé S.R. (2018). Preventing brain injury after cardiopulmonary bypass will require more than just dialing up the pressure. Circulation.

[11] Kiabi F.H., Soleimani A., Habibi M.R. (2019). Neuroprotective Effect of Low Mean Arterial Pressure on Postoperative Cognitive Deficit Attenuated by Prolonged Coronary Artery Bypass Time: A Meta-Analysis. Braz. J. Cardiovasc. Surg..

[12] Taylor K.M. (1998). Brain damage during cardiopulmonary bypass. Ann. Thorac. Surg..

[13] Baethge C., Goldbeck-Wood S., Mertens S. (2019). SANRA—A scale for the quality assessment of narrative review articles. Res. Integr. Peer Rev..

[14] Li Y., Walicki D., Mathiesen C., Jenny D., Li Q., Isayev Y., Reed J.F., Castaldo J.E. (2009). Strokes After Cardiac Surgery and Relationship to Carotid Stenosis. Arch. Neurol..

[15] Masabni K., Raza S., Blackstone E.H., Gornik H.L., Sabik J.F. (2015). Does preoperative carotid stenosis screening reduce perioperative stroke in patients undergoing coronary artery bypass grafting?. J. Thorac. Cardiovasc. Surg..

[16] Neumann F.J., Sousa-Uva M., Ahlsson A., Alfonso F., Banning A.P., Benedetto U., Byrne R.A., Collet J.P., Falk V., Head S.J. (2019). 2018 ESC/EACTS Guidelines on myocardial revascularization. Eur. Heart J..

[17] Narayan P., Khan W., Das D., Biswas R.G., Das M., Rupert E. (2017). Carotid artery screening at the time of coronary artery bypass—Does it influence neurological outcomes?. Int. J. Cardiol..

[18] Masabni K., Sabik J.F., Raza S., Carnes T., Koduri H., Idrees J.J., Beach J., Riaz H., Shishehbor M.H., Gornik H.L. (2016). Nonselective carotid artery ultrasound screening in patients undergoing coronary artery bypass grafting: Is it necessary?. J. Thorac. Cardiovasc. Surg..

[19] AbuRahma A.F., Avgerinos E.D., Chang R.W., Darling R.C., Duncan A.A., Forbes T.L., Malas M.B., Murad M.H., Perler B.A., Powell R.J. (2022). Society for Vascular Surgery clinical practice guidelines for management of extracranial cerebrovascular disease. J. Vasc. Surg..

[20] Fassiadis N., Adams K., Zayed H., Goss D., Deane C., MacCarthy P., Rashid H. (2008). Occult carotid artery disease in patients who have undergone coronary angioplasty. Interact. Cardiovasc. Thorac. Surg..

[21] Poorthuis M.H.F., Sherliker P., Morris D.R., Massa M.S., Clarke R., Staplin N., Lewington S., de Borst G.J., Bulbulia R., Halliday A. (2021). Development and Internal Validation of a Risk Score to Detect Asymptomatic Carotid Stenosis. Eur. J. Vasc. Endovasc. Surg..

[22] Gaudino M., Angiolillo D.J., Di Franco A., Capodanno D., Bakaeen F., Farkouh M.E., Fremes S.E., Holmes D., Girardi L.N., Nakamura S. (2019). Stroke after coronary artery bypass grafting and percutaneous coronary intervention: Incidence, pathogenesis, and outcomes. J. Am. Heart Assoc..

[23] Asta L., Falco D., Benedetto U., Porreca A., Majri F., Angelini G.D., Sensi S., Di Giammarco G. (2024). Stroke after Cardiac Surgery: A Risk Factor Analysis of 580,117 Patients from UK National Adult Cardiac Surgical Audit Cohort. J. Pers. Med..

[24] D’oria M., Mani K., DeMartino R., Czerny M., Donas K.P., Wanhainen A., Lepidi S. (2021). Narrative review on endovascular techniques for left subclavian artery revascularization during thoracic endovascular aortic repair and risk factors for postoperative stroke. Interact. Cardiovasc. Thorac. Surg..

[25] Jonsson K., Barbu M., Nielsen S.J., Hafsteinsdottir B., Gudbjartsson T., Jensen E.M., Silverborn M., Jeppsson A. (2022). Perioperative stroke and survival in coronary artery bypass grafting patients: A SWEDEHEART study. Eur. J. Cardiothorac. Surg..

[26] Tarakji K.G., Sabik J.F., Bhudia S.K., Batizy L.H., Blackstone E.H. (2011). Temporal onset, risk factors, and outcomes associated with stroke after coronary artery bypass grafting. JAMA.

[27] Magedanz E.H., Guaragna J.C.V.d.C., Albuquerque L.C., Wagner M.B., Chieza F.L., Bueno N.L., Bodanese L.C. (2021). Risk Score Elaboration for Stroke in Cardiac Surgery. Braz. J. Cardiovasc. Surg..

[28] Ding Q., Liu H., Zhang Z., Goldhammer J., Yuen E., Li Z., Yao L., Young N., Boyd D., Weintraub W. (2018). Perioperative aspirin and long-term survival in patients undergoing coronary artery bypass graft. Sci. Rep..

[29] Zaka A., Gupta A., Lombardo A., Kovoor J., Bacchi S., Smith J., Bennetts J., Maddern G. (2023). Perioperative aspirin and coronary artery bypass graft surgery: A meta-analysis of randomised controlled trials. Eur. Heart J..

[30] Jannati M. (2024). Risk factors for stroke post coronary artery bypass graft surgery: A review of literature. Med. Clínica Práctica.

[31] Ramponi F., Seco M., Brereton R.J.L., Gaudino M.F.L., Puskas J.D., Calafiore A.M., Vallely M.P. (2021). Toward stroke-free coronary surgery: The role of the anaortic off-pump bypass technique. J. Card. Surg..

[32] Lawton J.S., Tamis-Holland J.E., Bangalore S., Bates E.R., Beckie T.M., Bischoff J.M., Bittl J.A., Cohen M.G., DiMaio J.M., Writing Committee Members (2022). 2021 ACC/AHA/SCAI Guideline for Coronary Artery Revascularization: A Report of the American College of Cardiology/American Heart Association Joint Committee on Clinical Practice Guidelines. Circulation.

[33] Dominici C., Dominici C., Salsano A., Salsano A., Nenna A., Nenna A., Spadaccio C., Spadaccio C., El-Dean Z., El-Dean Z. (2019). Neurological outcomes after on-pump vs off-pump CABG in patients with cerebrovascular disease. J. Card. Surg..

[34] Patel N., Banahan C., Janus J., Horsfield M.A., Cox A., Marshall D., Colman J., Morlese J., Evans D.H., Hannon C. (2020). Neurological impact of emboli during adult cardiac surgery. J. Neurol. Sci..

[35] Preoperative Transcranial and Carotid Doppler Study in Coronary Artery Bypass Graft Patients—PubMed. [Online]. https://pubmed.ncbi.nlm.nih.gov/21857617/.

[36] Pierik R., Zeillemaker-Hoekstra M., Scheeren T.W., Erasmus M.E., Luijckx G.-J.R., Rienstra M., Uyttenboogaart M., Nijsten M., Bergh W.M.v.D. (2022). Early Thromboembolic Stroke Risk of Postoperative Atrial Fibrillation Following Cardiac Surgery. J. Cardiothorac. Vasc. Anesth..

[37] Mrkobrada M., Chan M.T.V., Cowan D., Campbell D., Wang C.Y., Torres D., Malaga G., Sanders R.D., Sharma M., Brown C. (2019). Perioperative covert stroke in patients undergoing non-cardiac surgery (NeuroVISION): A prospective cohort study. Lancet.

[38] Tsukagoshi J., Yokoyama Y., Fujisaki T., Takagi H., Shirasu T., Kuno T. (2023). Systematic review and meta-analysis of the treatment strategies for coronary artery bypass graft patients with concomitant carotid artery atherosclerotic disease. J. Vasc. Surg..

[39] Knipp S.C., Holst T., Bilbilis K., von Velsen O., Ose C., Diener H.-C., Jakob H., Ruhparwar A., Jöckel K.-H., Weimar C. (2022). Five-Year Results of Coronary Artery Bypass Grafting with or without Carotid Endarterectomy in Patients with Asymptomatic Carotid Artery Stenosis: CABACS RCT. Stroke.

[40] Borger M.A., Ivanov J., Weisel R.D., Rao V., Peniston C.M. (2001). Stroke during coronary bypass surgery: Principal role of cerebral macroemboli. Eur. J. Cardio-Thorac. Surg..

[41] Ye J., Webb J.G. (2014). Embolic capture with updated intra-aortic filter during coronary artery bypass grafting and transaortic transcatheter aortic valve implantation: First-in-human experience. J. Thorac. Cardiovasc. Surg..

[42] Willard R., Scheinerman J., Pupovac S., Patel N.C. (2024). The Current State of Hybrid Coronary Revascularization. Ann. Thorac. Surg..

[43] Hirai T., Kitahara H., Balkhy H.H., Blair J.E. (2019). Advanced Hybrid Complete Revascularization with TECAB and Impella-Assisted PCI of CTO. Cardiovasc. Revasc. Med..

[44] Naito S., Demal T.J., Sill B., Reichenspurner H., Onorati F., Gatti G., Mariscalco G., Faggian G., Santini F., Santarpino G. (2022). Neurological Complications in High-Risk Patients Undergoing Coronary Artery Bypass Surgery. Ann. Thorac. Surg..

[45] Kowalewski M., Pawliszak W., Malvindi P.G., Bokszanski M.P., Perlinski D., Raffa G.M., Kowalkowska M.E., Zaborowska K., Navarese E.P., Kolodziejczak M. (2016). Off-pump coronary artery bypass grafting improves short-term outcomes in high-risk patients compared with on-pump coronary artery bypass grafting: Meta-analysis. J. Thorac. Cardiovasc. Surg..

[46] Bonacchi M., Prifti E., Frati G., Leacche M., Giunti G., Proietti P., Salica A., Papalia U. (2002). Concomitant carotid endarterectomy and coronary bypass surgery: Should cardiopulmonary bypass be used for the carotid procedure?. J. Card. Surg..

[47] Squizzato F., Xodo A., Taglialavoro J., Zavatta M., Grego F., Antonello M., Piazza M. (2021). Early outcomes of routine delayed shunting in carotid endarterectomy for symptomatic patients. J. Cardiovasc. Surg..

[48] Xodo A., Barbui F., Desole A., Pilon F., Zaramella M., Milite D. (2023). Bypass and other modified reconstruction techniques for ‘challenging’ carotid cases: A comparison with conventional endarterectomy. Vascular.

[49] Paraskevas K.I., Nduwayo S., Saratzis A.N., Naylor A.R. (2017). Carotid Stenting Prior to Coronary Bypass Surgery: An Updated Systematic Review and Meta-Analysis. Eur. J. Vasc. Endovasc. Surg..

[50] Williams Z., Olivere L.A., Gilmore B., Weissler H., Cox M.W., Long C., Shortell C.K., Schroder J., Southerland K.W. (2020). Safety and Feasibility of Simultaneous Transcarotid Revascularization with Flow Reversal and Coronary Artery Bypass Grafting for Concomitant Carotid Artery Stenosis and Coronary Artery Disease. Vasc. Endovasc. Surg..

[51] Staged Carotid Artery Stenting and Coronary Artery Bypass Surgery Versus Isolated Coronary Artery Bypass Surgery in Concomitant Coronary and Carotid Disease. [Online]. https://www.hmpgloballearningnetwork.com/site/jic/articles/coronary-artery-bypass-graft-surgery-patients-receiving-antiplatelet-therapy-can-we-fine-t.

[52] Rodino W., Panetta T.F., Burack J.H., Bryan D.H., Williams R.F. (1996). Combined carotid endarterectomy, innominate artery reconstruction, and coronary artery bypass grafting: Case report. J. Vasc. Surg..

[53] Antonello M., Xodo A., Squizzato F., Zavatta M., Maturi C., Piazza M. (2022). Preliminary experience with new generation balloon expandable stent-graft in the treatment of innominate artery obstructive disease. J. Cardiovasc. Surg..

[54] Dzierwa K., Pieniazek P., Musialek P., Piatek J., Tekieli L., Podolec P., Drwila R., Hlawaty M., Trystula M., Motyl R. (2011). Treatment strategies in severe symptomatic carotid and coronary artery disease. Med. Sci. Monit..

[55] Likosky D.S., Marrin C.A., Caplan L.R., Baribeau Y.R., Morton J.R., Weintraub R.M., Hartman G.S., Hernandez F., Braff S.P., Charlesworth D.C. (2003). Determination of etiologic mechanisms of strokes secondary to coronary artery bypass graft surgery. Stroke.

